# Competency-based Standard Setting for a High-stakes Objective Structured Clinical Examination (OSCE): Validity Evidence

**DOI:** 10.15694/mep.2018.0000200.1

**Published:** 2018-09-06

**Authors:** Ming Lee, Estebes Hernandez, Rachel Brook, Edward Ha, Christina Harris, Monica Plesa, Daniel Kahn

**Affiliations:** 1 David Geffen School of Medicine at UCLA

**Keywords:** Standard Setting, Objective Structured Clinical Examination, OSCE, Validity, Modified Angoff Method, Clinical Competency Assessment

## Abstract

This article was migrated. The article was marked as recommended.

Introduction

Medical educators need to demonstrate that their trainees meet expected competency levels when progressing through medical education. This study aimed to develop competency-based pass/fail cut-scores for a graduation required Objective Structured Clinical Examination (OSCE), and examine validity evidence for new standards.

Methods

Six clinicians used the modified Angoff method to determine the cut-scores for an 8-station OSCE. The clinicians estimated the percentage of minimally competent students who would answer each checklist item correctly. Inter-rater reliability, differences in other academic achievements between pass/fail groups, educational impact, and response process were examined.

Results

One hundred seventy-four rising 4th-year medical students participated in the OSCE. The cut-scores determined for the OSCE resulted in a substantially lower failure rate (5% vs. 29% of the previous year). The inter-rater reliability across domains and cases was .98 (95% CI = .97 - .99). The pass/fail groups significantly differed in six of the eight measures of academic achievements included in the study.

Discussion

The impact of the standards setting was substantial as it significantly reduced the failure rate and burdens of remediation for both students and faculty. The very high inter-rater reliability indicates that the modified Angoff method produced reliable cut-scores. The significant differences between the pass/fail groups in other measures support external validity of the standards and ensure no false passes. The study also supports response process validity by including discussion among judges and check of previous student performances, as well as recruiting and training multiple clinician educators experienced in medical student teaching.

Conclusion

Findings of the study provide strong evidence supporting validity of the new cut-scores from a wide spectrum of validity metrics, including response process, internal structure, relations to other variables, and consequences. The study also added to the literature the value of the modified Angoff method in determining competency-based standards for OSCEs.

## Introduction

The paradigm in medical education has shifted from a structure- and process-based model suggested by Flexner (1910) to a competency-based framework that arose at the turn of the 21 ^st^ century in response to a call for accountability and responsibility from medical institutions to train competent physicians ( Carraccio et al., 2002). As a result, the US Accreditation Council of Graduate Medical Education (ACGME) launched its competency frameworks and recent outcomes-based Milestones Project for postgraduate medical training (ACGME, [2017]). The Association of American Medical Colleges (AAMC) also acted to link clinical competencies expected from medical students to 13 so-called entrustable professional activities (EPAs) ( AAMC, 2014), in which medical graduates are capable of delivering independent practice without direct supervision upon entering residency. These new initiatives have driven medical institutions to revisit their existing educational practices from curricular design, delivery of teaching, to assessment of learning outcomes and evaluation of program effectiveness.

Although medical institutions bear social and accreditation accountability in training clinically competent graduates for professional practice, the assessment of clinical performance is not only complex but also controversial ( Martin and Jolly, 2002). Clinical competence is generally considered multifaceted, encompassing diverse attributes ranging from information acquisition ability and application of basic and clinical knowledge, to healthcare problem solving, communication skills, and personal characteristics ( Lee and Wimmers, 2011). Among various assessment tools of clinical competencies, Objective Structured Clinical Examination (OSCE) has been the most commonly used one since the 1980s ( Hauer et al., 2005). The tool has since received varying psychometric evaluations and methodological criticism ( Hodges et al., 1999; Hodges, 2003; Patricio et al., 2013; Dong et al., 2014; White et al., 2008). Although its value and use is still controversial, it is generally believed to be a reliable and feasible instrument for measuring clinical skills.

An outstanding challenge for OSCEs is setting the minimum standard, or passing mark ( Smee and Blackmore, 2001; Boursicot and Roberts, 2006). Traditionally, there are two types of standards for grade decisions: (1) relative standards, which are norm-referenced as the standards are based on the performance of a certain group of examinees, such as a mean score of a test, and (2) absolute standards, which are criterion-referenced as the standards are based on desired levels of mastery, such as a score of 60% correctness. Which type of standards is to be used depends on the purpose of the test. Absolute standards have been preferred and are believed more appropriate for OSCEs in a competency-based assessment system since the purpose is to compare examinees’ performances with predefined outcomes ( Norcini, 2003; Pereira et al., 2018). A variety of standard setting methods has been developed and reported in the literature to determine the passing mark for OSCEs ( Norcini, 2003; De Champlain, 2014). Among them, the modified Angoff method is believed to be used and studied most often ( De Champlain, 2014; Jalili et al., 2011; Cizek, 2012), and is frequently applied in licensing and certifying settings ( Norcini, 2003). The modified Angoff method uses expert judges to estimate the percentage of minimally competent examinees who will succeed on each item being assessed ( Angoff, 1971). The judges may discuss and change their estimates, and are informed by the performance of prior examinees, a process called “reality check” ( Jaeger, 1978; Norcini, 1988). The estimates are averaged across items, cases, and judges to come up with absolute cut-scores, the standards.

Our school has been using OSCEs for more than two decades to assess trainees’ clinical performances across all levels of medical education. An eight-station OSCE, called Clinical Performance Examination (CPX), has been administered to rising final year students since 1996. The CPX cases, checklists, and standardized patient (SP) training materials have been collaboratively developed by the California Consortium for the Assessment of Clinical Competence (CCACC), which consists of our school and seven other public and private medical schools across the State of California. Students spend fifteen minutes at each station to conduct a focused work-up on a trained SP, who records students’ clinical performances based on a case-specific checklist. The scores are averaged across the items of three individual domains - History Taking (HX), Physical Examination (PE), and Physician-Patient Interaction (PPI) - and eight cases to form percent correct domain scores, and an average of the three domain scores form the overall score. The standards before 2010 were 2 standard deviations below the means of the three domain scores and the overall score, resulting in an average failure rate around 8%. These norm-referenced standards were later considered too lenient and changed in a rather arbitrary manner to criterion-referenced standards of 60% on the three domains (58% was used for PE in 2013 and 2015) and 65% on Overall between 2011 and 2015, resulting in a jump in the failure rate to nearly 30% for most of these years. This dramatic change inevitably brought student complaints and heavy remediation workloads on faculty, and subsequently led to a decision to re-set the standards by using a well-studied, popularly accepted standard setting method. We conducted this study with a threefold purpose, including to (1) determine the cut-scores for the three CPX domains and the overall by using the modified Angoff method; (2) examine validity evidence for the derived standards by using the conceptual framework for test validation jointly developed by the American Educational Research Association (AERA), American Psychological Association (APA), and the National Council on Measurement in Education (NCME) (2014); and (3) evaluate the value of the modified Angoff method in setting standards for OSCEs. Given the pervasive use of OSCEs in medical institutions around the world, we expect the findings of the study to be of value for other institutions in their decision-making when setting standards for OSCEs.

## Methods

### Procedure

Six clinician educators (EH, RB, EHa, CH, MP, and DK) were recruited for the study based on their prior experience in supervising medical students at clinical sites. They were first introduced to the purpose of the standard setting task and the CPX, differences between norm- and criterion-referenced standards in grade decisions, and the modified Angoff method by a medical educator (ML) specialized in educational assessment and CPX administration. The group was then engaged in a discussion about the definition of minimal competency in clinical performance for rising final year medical students. The clinicians were encouraged to take as examples those students whom they encountered at clinical sites and would not feel comfortable to pass or fail them without further consideration or additional evidence. They then worked together to estimate the percentages of minimally competent students who would perform accurately on five checklist items (at least one item from each of the HX, PE, and PPI domains) from a case included in the 2016 CPX. After this initial training session, the clinicians individually completed the estimates of the remaining items of the case. The group was reconvened at the next meeting to review the means and ranges of the estimates made by them for each item, discuss individual rationales for the estimates, learn the item performance of a prior cohort of rising final year students on the same case (“reality check”), and adjust their estimates where needed. The means and ranges of the second estimation round were calculated afterwards for another review and adjustment, if desired. The same procedure was repeated for the other seven cases used in the 2016 CPX. Final estimates were averaged across clinicians and cases to obtain the cut-scores for the three domains (HX, PE & PPI) and the Overall (an average of the three domains).

### Measures

We used nine assessment measures in the study to examine the relations of the cut-scores to other variables (external validity), including: (1) United States Medical Licensing Examination (USMLE) Step 1; (2) USMLE Step 2 Clinical Knowledge (CK); (3) a 3-station Objective Structured Clinical Examination (OSCE) administered at the end of the second year; (4) National Board of Medical Examiners (NBME) Internal Medicine (IM) subject examination; (5) NBME Family Medicine (FM) subject examination; (6) IM clerkship rating; (7) FM clerkship rating; (8) Ambulatory Medicine (AM) clerkship rating; and (9) USMLE Step 2 Clinical Skills (CS) grade.

Both the USMLE Step 1 and Step 2 CK use multiple-choice questions to assess knowledge acquisition and application in, respectively, basic sciences and clinical sciences. The 2 ^nd^-year OSCE produced a percent correct score averaged across three domains (HX, PE, PPI) and three cases using faculty panel-created checklists. The IM, FM, and AM clerkship ratings were average scores given by preceptors using a five-point Likert scale, with higher scores indicating more satisfactory performance, to evaluate students’ performances in nine areas: history taking, physical examination, oral case presentation, write-ups and progress notes, fund of knowledge, clinical judgment, physician-patient interaction, professional attitudes and behaviors, and overall rotation performance. The USMLE Step 2 CS is a 12-station OSCE, with a 15-minute SP encounter followed by a 10-minute patient note write-up for each station. The exam is scored based on three subcomponents: Communication and Interpersonal Skills (CIS), Spoken English Proficiency (SEP), and Integrated Clinical Encounter (ICE), but only a pass or fail grade is reported. The recommended minimum passing level for the CS, and other USMLE exams, is reviewed periodically (typically every 3-4 years) ( USMLE, 2017).

### Data Analysis

We calculated intra-class correlation coefficients (ICCs), using two-way mixed effects model and absolute agreement type, to examine inter-rater reliability. Paired t-tests were conducted to compare differences between initial and final estimations by domain to examine rater training effect, an indication of response process validity. We calculated Pearson correlation coefficients between cut-scores and difficulty levels (percentages of students who answered correctly) by case and domain to examine internal structure validity. To examine the relations to other variables (external validity) and ensure no false passes, we conducted analysis of variance (ANOVA) on the differences in the eight of nine major medical school achievements, except the Step 2 CS, among three groups of the students (passed by both old and new standards, failed by both standards, and those who would have failed in the old standards but passed in the new standards). For the Step 2 CS, Pearson chi-square was calculated to examine the relationship between its grade and the grade of the CPX. For each of the home-made assessments (#3-8 measures listed above) as well as the 2016 CPX, we calculated the internal consistency reliability coefficients (Cronbach’s alphas) to examine reliability of the scores produced by the individual assessments.

## Results/Analysis

The six clinicians were equally spilt by gender. Five of them were internists, and one was family medicine physician. A total of 174 rising final year medical students participated in the 2016 CPX upon which the standard setting was conducted and validated.

The inter-rater reliability coefficients ranged from .89 to .98 for each of the three domains (HX, PE, and PPI), and .97 to .99 for each of the eight cases, resulting in an overall inter-rater reliability across domains and cases of .98 (95% Confidence Interval = .97 - .99). Cronbach’s alphas for the five home-made assessments, including the 2 ^nd^-year OSCE, 2016 CPX, and IM, FM, and AM clerkship evaluations, were .79, .80, .93, .93, and .94, respectively, indicating good to very high levels of internal consistency reliability for these assessments.

Table 1 shows the discrepancies between the initial and final estimations averaged across the eight cases for each domain. The initial estimation was done independently by each clinician before group discussion and reality check. None of the discrepancy, except that in the Physician-Patient Interaction (PPI) domain (Δ = -4.75, t = -4.18, p < .01), was statistically significant, showing a likely training effect on the clinicians’ initial estimation. An overall Pearson correlation coefficient of .78 (p < .001) was found between domain-by-case difficulties and corresponding cut-scores, indicating the clinicians accounted for the difficulty level of each case domain in their estimation.

**Table 1.** Discrepancies between initial and final estimations of cut-scores ^*^

Domain	Initial Estimation	Final Estimation	Difference	t-test	p-value
History Taking	60.99	60.06	0.93	.70	.52
Physical Exam	51.43	47.54	3.90	2.22	.08
Physician-Patient Interaction	57.77	62.52	-4.75	-4.18	.01
Overall	56.73	56.71	0.03	.03	.98

^*^ The cut-score for each domain is an average of estimated percentages of minimally competent students who would perform correctly on the items of the domain.

The cut-scores of the 2016 CPX determined via the modified Angoff method, the cut-scores used for the 2015 CPX, and their associated failure rates are presented in Table 2. The total failure rates were 29% (N = 56) and 5% (N = 8) for the 2015 and 2016 CPX, respectively, showing a substantial impact on the reduction of failure rate resulted from the standard setting exercise.

**Table 2.** Comparisons of cut-scores and failure rates between the 2015 and 2016 Clinical Performance Examination (CPX)

Domain	2015 CPX		2016 CPX	
	Cut-Score (% correct)	Failure Rate ^1^ N (%)	Cut-Score (% correct)	Failure Rate ^2^ N (%)
History Taking	60	6 (3%)	60	5 (3%)
Physical Exam	58	52 (27%)	48	5 (3%)
Physician-Patient Interaction	60	3 (2%)	63	0
Overall	65	9 (5%)	57	0

^1^ The total failure rate of the 2015 CPX is 29% (N = 56).

^2^ The total failure rate of the 2016 CPX is 5% (N = 8).

**Figure 1.**
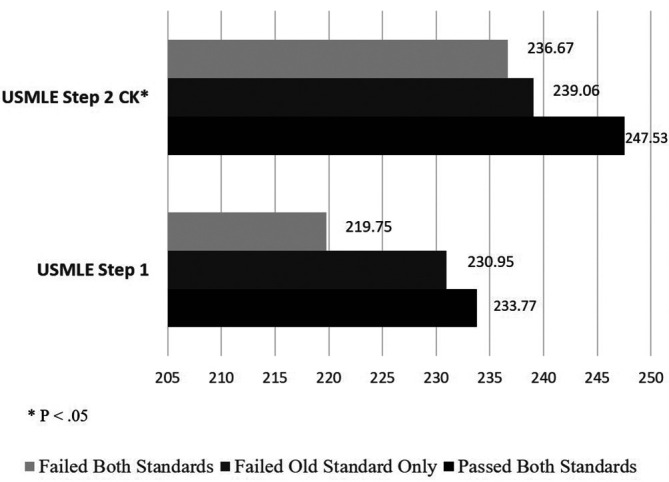
Comparison of the performance of Clinical Performance Examination (CPX) pass/fail groups determined by the 2015 and 2016 standards on USMLE Step 1 and Step 2 Clinical Knowledge (CK) Abbreviations: USMLE = United States Medical Licensing Examination

**Figure 2.**
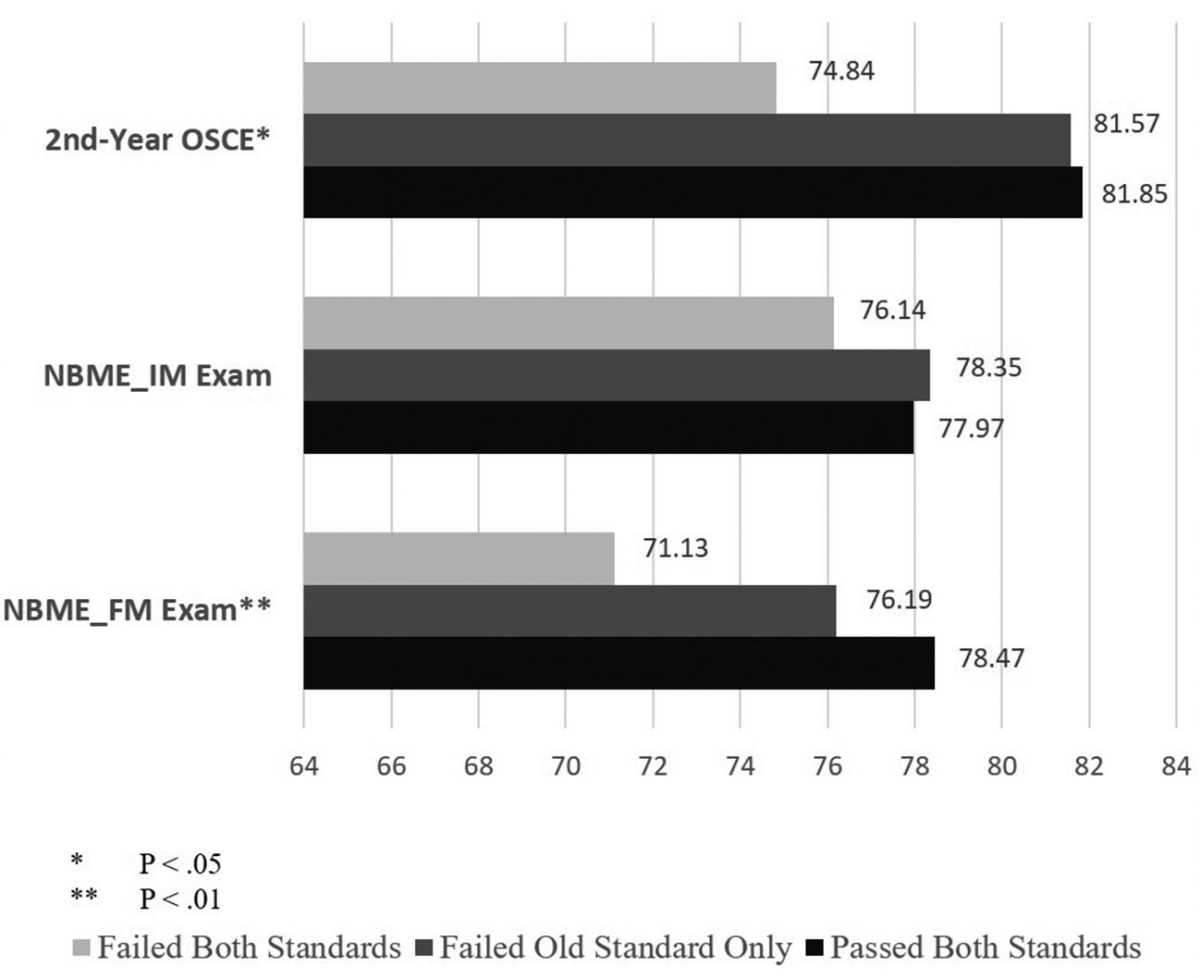
Comparison of the performance of Clinical Performance Examination (CPX) pass/fail groups determined by the 2015 and 2016 standards on 2nd-year OSCE and NBME_IM and NBME_FM subject exams Abbreviations: OSCE = Objective Structured Clinical Examination; NBME = National Board of Medical Examiners; IM = Internal medicine; FM = Family medicine

**Figure 3.**
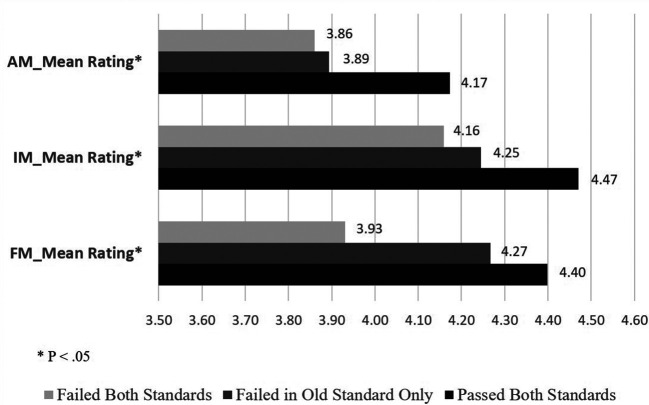
Comparison of the performance of Clinical Performance Examination (CPX) pass/fail groups determined by the 2015 and 2016 standards on Three Clerkship Ratings Abbreviations: AM = Ambulatory Medicine; IM = Internal medicine; FM = Family medicine

When examining external validity by comparing the other academic achievements among the three groups of the students - those who were determined passed or failed by both the 2015 and 2016 CPX standards and those who would have failed by the 2015 standards but passed by the 2016 standards - we found the three groups were significantly different in the 2 ^nd^-year OSCE (F(2,149) = 3.27, P < .05), USMLE Step 2 CK (F(2,146) = 3.16, P < .05), NBME Family Medicine subject exam (F(2,169) = 5.06, P < .01), and the mean clerkship ratings for IM (F(2,168) = 4.35, P < .05), FM (F(2,168) = 3.62, P < .05), and AM (F(2,168) = 4.12, P < .05) (see Figures 1- 3). The post-hoc analyses showed that the significant differences in all of these comparisons were between the pass and fail groups, with the group failing only by the old standards performing similarly to the pass group except in the AM clerkship rating. The findings support the relationship of the new standards with other related educational measures as well as no false passes of the CPX by new standards.

Table 3 shows the findings of the relationship between the CPX grade, by using both the 2015 and 2016 standards, and the USMLE Step 2 Clinical Skills (CS) grade. Among the 170 students who took the CS and received a grade, 163 (95.9%) passed and 7 (4.1%) failed the exam. Among those who passed the CS, 157 (96.3%) students also passed the CPX by the new standards, compared with only 137 (84%) students who would have passed the CPX if using the old standards. Among the 7 students who failed the CS, only 1 (14.3%) of them also failed the CPX by the new standards, compared with 2 (28.6%) who would have failed the CPX if using the old standards. The results indicated that, in comparison with the old standards, the new standards did not impose an unnecessary burden of going through remediation activities on those 20 students who would eventually pass the clinical skills board exam, and only mis-identified one at-risk student for the CS. The Pearson chi-square test for the relationship between the CPX grade determined by the new 2016 standards and the CS grade, however, was not statistically significant (χ ^2^ (1, N = 170) = 1.91, p = .17), indicating a lack of significant relationship between the two grades.

**Table 3.** Comparison of the performance of Clinical Performance Examination (CPX) pass/fail groups determined by the 2015 and 2016 standards on the grade of USMLE Step 2 Clinical Skills (CS)

			CPX Grade			Total
			Passed by BothStandards	Failed by 2015 Standards Only	Failed by Both Standards	
Step2CS Grade	Fail	Count	5	1	1	7
		% within Step2CS Grade	71.4%	14.3%	14.3%	100.0%
	Pass	Count	137	20	6	163
		% within Step2CS Grade	84.0%	12.3%	3.7%	100.0%
Total		Count	142	21	7	170
		% within Step2CS Grade	83.5%	12.4%	4.1%	100.0%

## Discussion

As medical education has shifted to a competency-based system, assessment practices that can be trusted in identifying competent trainees at different levels of educational trajectory need to be in place. Any high-stakes assessments that lead to important decisions in processes, such as admission and promotion, demand especially careful consideration in setting pass/fail standards. This study examined validity evidence for a set of new standards, derived from the modified Angoff method, of a high-stakes, graduation-required Objective Structured Clinical Examination (OSCE), called Clinical Performance Examination (CPX). The new standards determined for the History Taking (HX), Physical Examination (PE), and Physician-Patient Interaction (PPI) domains, and the Overall were 60%, 48%, 63%, and 57%, respectively. These cut-scores, compared with the ones used in the prior year (60%, 58%, 60%, 65%, respectively), were either the same (HX), lower (PE and Overall), or higher (PPI), showing the discrepancies between arbitrarily defined old standards and the new ones derived from the formal procedure of a well-researched and longstanding standard setting method ( Cizek, 2012; De Champlain, 2014; Norcini, 2003). Findings of the study provided strong evidence supporting all aspects of validity of the new standards using the educational testing and assessment framework developed by the American Educational Research Association (AERA), American Psychological Association (APA), and the National Council on Measurement in Education (NCME) (2014).

In regard to **response process validity**, six clinician educators were recruited for the standard setting task, each of them having had extended experience in teaching and supervising medical students at clinical sites. The findings demonstrating mostly insignificant discrepancies between clinician judges’ initial and final estimations of minimally competent performance indicate efficacy of training and the likely effect of the clinicians’ experiences in teaching, both of which prepared the clinician judges well for the task. The very high inter-rater reliability provides additional support for training efficacy, which also supports **internal structure validity** of the cut-scores. The finding of an overall high correlation between case-domain difficulties and cut-scores provides further support for internal structure validity, indicating the cut-scores were determined by accounting for case and domain specificity and difficulty. For validity examined through **relations to other variables (external validity)**, the significant differences found among the three pass/fail groups (passed or failed both standards, and failed in old standards but passed in new standards) in six of the eight other academic assessments are evidence supporting the validity. The findings also show that no false passes of the CPX were granted to incompetent students as the students who would have failed by old standards but passed by new standards did not significantly differ from the students who passed by both standards in the majority of the other assessments included in the study. The most impressive finding is that the new standards significantly reduced the failure rate of the 2016 CPX from 29% (N = 56) the prior year to 5% (N = 8), showing a strong support for **consequential (impact) validity**. The reduced failure rate seems justified by the finding that 20 students who would have failed the CPX by the old standards, but passed by the new standards, subsequently passed the Step 2 CS. The reduction in failure rate led to reduced burden of remediation works for both students and faculty, allowed faculty more time and focused efforts on the students who truly need remediation, and avoided unwarrantedly imposing stress and humiliation of failure to students.

All the findings of this study that provide validity evidence for the CPX cut-scores also demonstrate the value of the modified Angoff method in producing reliable and valid cut-scores for OSCEs. The method produces criterion-referenced pass/fail standards that are deemed desired for clinical performance assessments in competency-based medical education. By allowing the judges to modify their estimates after general discussion and reality check, the method gauges their initial judgements and inter-rater reliability of the cut-scores. The high inter-rater reliability is also likely a result of the Angoff method which allows for summating and averaging the estimated probabilities across the items for each domain and case ( Boursicot and Roberts, 2006).

There are a few limitations to the study. Firstly, the study was conducted in a single medical school. Although the findings may not be replicable in other institutions using different OSCE materials or providing different protocols of training to clinician judges, our findings add to the existing evidence supporting the modified Angoff method for setting standards for OSCEs. Secondly, the Angoff method requires estimations of the probability of success by hypothetical examinees (minimally competent students), a task deemed unrealistic, even for experts, by critics ( Smee and Blackmore, 2001; Shepard, 1995). The majority (4 of the 6) of our clinician judges also felt challenged in determining what constituted minimal proficiency for rising final year medical students ( Hernandez et al., 2017). The very high inter-rater reliability and external validity of the cut-scores found in the study, however, show that the seemingly challenging task could perhaps be overcome by recruiting experienced clinician judges, providing sufficient training, and giving judges an opportunity to discuss among themselves and modify estimates. Several other studies have also shown the possibility of making consistent item performance estimates within and across judges using the Angoff method ( Hambleton et al., 2000; Plake et al., 2000). Thirdly, the cut-scores derived in the study failed to identify seven of the eight at-risk students who subsequently failed the USMLE Step 2 CS. Given that one of the purposes of the CPX is to prepare our students for taking the CS, this lack of advance warning might lead the students and faculty to miss a timely remediation opportunity. Although the detailed scoring method and passing standards of the CS have never been released, the CPX is known to be scored differently from the CS, given the fact that the CS includes patient note write-up and spoken English proficiency in the scoring whereas the CPX does not. The student performance outcomes on the two exams may also be expected to be different given that the CS is a 12-station OSCE, compared with an 8-station CPX, which may entail more fatigue and higher anxiety than the CPX. In addition, a change to increase the passing level for all three subcomponents of the Step 2 CS taken on or after September 10, 2017 ( USMLE, 2017) affected three of the seven students who passed the 2016 CPX but took the CS in late 2017 and failed. All these factors may contribute to a lack of relationship between the CS and CPX grades, which may not be directly affected by the new CPX standards.

## Conclusion

This study followed the procedure of the modified Angoff method to set new passing marks for a high-stakes OSCE. The new standards substantially reduced the failure rate with no indication of compromising the integrity of pass/fail decisions. Findings of the study provide strong evidence supporting validity of the new cut-scores from a wide spectrum of validity metrics, including response process, internal structure, relations to other variables, and consequences. The study also added to the literature the value of the modified Angoff method in determining competency-based standards for OSCEs.

## Take Home Messages

• In the era of competency-based medical education, medical educators need to determine whether their trainees meet expected competency levels when progressing through the training.
• The modified Angoff method, supplemented by discussion among judges and reality check, can produce reliable and valid cut-scores for OSCEs.
• Validated competency-based standards may prevent passing incompetent students or burdening competent students with unwarranted remediation works.
• Recruiting and training multiple experienced clinician educators as judges is essential for setting standards for OSCEs using the modified Angoff method.
• Any high-stakes exams demand careful consideration for setting criterion-referenced pass/fail standards.

## Notes On Contributors

Dr. Ming Leeis professor of Medical Education, and co-director of Medical Education Fellowship at Dean’s Office, David Geffen School of Medicine at UCLA, Los Angeles, California.

Dr. Estebes Hernandezis assistant clinical professor, Department of Medicine, and co-director, Clinical Performance Examination, David Geffen School of Medicine at UCLA, Los Angeles, California.

Dr. Rachel Brookis assistant clinical professor, Department of Medicine (DOM), director of DOM ambulatory medicine clerkship, and curricular lead for Doctoring 1, David Geffen School of Medicine at UCLA, Los Angeles, California.

Dr. Edward Hais associate clinical professor, Department of Medicine, and assistant dean for Curricular Affairs: Clinical Education, David Geffen School of Medicine at UCLA, Los Angeles, California.

Dr. Christina Harrisis assistant clinical professor, Department of Medicine, VA Greater Los Angeles Healthcare System, and associate program director, Internal Medicine Residency Program, David Geffen School of Medicine at UCLA, Los Angeles, California.

Dr. Monica Plesais assistant clinical professor, Department of Family Medicine, and director of family medicine clerkship at UCLA-Santa Monica Family Health Center, David Geffen School of Medicine at UCLA, Los Angeles, California.

Dr. Daniel Kahnis assistant clinical professor, Department of Medicine, and co-director, Clinical Performance Examination, David Geffen School of Medicine at UCLA, Los Angeles, California.
