# Supplementary material for: Competency-based Standard Setting for a High-stakes Objective Structured Clinical Examination (OSCE): Validity Evidence
Source: MedEdPublish (2016). 2018 Sep 6;7:200. [Version 1] doi: 10.15694/mep.2018.0000200.1 (PMC10701848; doi:10.15694/mep.2018.0000200.1)
Supplement: Peer review history

# Reviewer report: Reviewer response for version 1

**DOI:** 10.21956/mep.19669.r29369

**Published:** 2018-09-21

**Authors:** Leila Niemi-Murola

**Affiliations:** 1 University of Helsinki

This review has been migrated. The reviewer awarded 4 stars out of 5 This is an interesting article focusing on standard setting in high stakes OSCEs. Reliability and the validity of the cut-off score in practice is not always an easy task. The authors give a thorough description of a process when six clinician educators constructed the cut-off score. The clinicians were obviously not familiar with the Angoff method prior to the study. The authors used nine measures in the study to examine the relations of the cut-scores to other variables. The results was a high inter-rater reliability. This paper presents a feasible model of setting cut-off scores for educators striving to increase reliability and validity of their examinations. Clinicians are used to evaluate their students’ performance, but is has been challenge to construct assessment having sufficient rigor.

Reviewer Expertise:

NA

No decision status is available

# Reviewer report: Reviewer response for version 1

**DOI:** 10.21956/mep.19669.r29368

**Published:** 2018-09-21

**Authors:** Peter Dieter

**Affiliations:** 1 AMSE

This review has been migrated. The reviewer awarded 4 stars out of 5 The paper reports about a competency-based setting for an 8-station OSCE and the determination of cut-scores. This is a very important issue and so far -to my knowledge- no agreed solution has been determined. They study adds significant new data in this field.

Reviewer Expertise:

NA

No decision status is available
